# Recurrence Rate After Post-Operative Two-Hour Continuous Bladder Irrigation for Primary Non-Muscle-Invasive Bladder Cancer: A Retrospective Cohort Study

**DOI:** 10.3390/jpm16040175

**Published:** 2026-03-24

**Authors:** Patrick Sterner, Sanna Gimbergsson, Markus Johansson, Farhood Alamdari, Amir Sherif, Abbas Chabok, Johan Styrke

**Affiliations:** 1 Centre for Clinical Research Västmanland, Uppsala University, 72189 Västerås, Sweden; 2 Department of Urology, Västmanland Hospital Västerås, 72189 Västerås, Sweden; 3 Department of Diagnostics and Intervention, Urology and Andrology, Umeå University, 90187 Umeå, Sweden; 4 Department of Urology, Sundsvall Hospital, 85643 Sundsvall, Sweden; 5 Department of Surgery, Danderyd Hospital, Region Stockholm, 18288 Danderyd, Sweden

**Keywords:** non–muscle-invasive bladder cancer, urinary bladder neoplasms, local neoplasm recurrence, therapeutic irrigation, personalized treatment, retrospective studies, non-randomized controlled trials

## Abstract

**Background**: High recurrence rates for non-muscle-invasive bladder cancer (NMIBC) remain a clinical challenge. Recommended post-operative treatments are underutilized, highlighting the need for alternative strategies. Given the variability in bladder cancer prognosis, personalized treatment approaches are highly relevant. In this study, we evaluated post-operative two-hour continuous sterile water bladder irrigation (CSWBI) regarding recurrence and safety, as a potential addition to the treatment arsenal for bladder cancer. **Method**: In 2018, two-hour CSWBI was implemented as routine treatment after all transurethral resection procedures of the bladder (TURB), at the urology department of Sundsvall Hospital. All patients who underwent TURBs four years prior (control group) and four years after the implementation of CSWBI (intervention group) were analyzed. Primary NMIBC were included, MIBC and CIS were excluded. Data were collected retrospectively from patient records, including baseline characteristics, adverse events, and recurrence rates within 12 months follow-up. Statistical analyses included Chi-squared test, Wilcoxon rank-sum test, univariate and multivariate logistic regression analyses, Kaplan–Meier curves and log-rank test. **Results**: A total of 168 patients were included (control group n = 90, irrigation group n = 78). Median age was 73 years, 23% were female, 77% were male, and 74% were active or previous smokers. The recurrence rate within twelve months for the intervention group vs. the control group was: 27% vs. 21% (*p* = 0.4) respectively. CSWBI had no statistically significant impact on recurrence (OR 1.25, 95% CI 0.58–2.68, *p* = 0.6). Adverse effects were limited and equal between groups. **Conclusions**: Post-operative two-hour CSWBI did not significantly reduce NMIBC recurrence within twelve months in this cohort.

## 1. Introduction

The burden of high recurrence rates remains one of the main challenges regarding the treatment of patients who suffer from non-muscle-invasive bladder cancer (NMIBC). The two year recurrence rate is 10–40% and the five-year recurrence rate is up to 60% [1,2,3,4,5]. This requires ambitious standards for surveillance with frequent monitoring, leading to physical and psychological stress for the patients and an economic burden on the health care system.

Offering patients personalized treatment approaches depending on clinical risk factors, perioperative considerations and comorbidity is a clinical matter of course. The Swedish and European (EAU) guidelines recommend a single, immediate, post-operative intravesical instillation of chemotherapy (SI) to decrease recurrences for patients with low- or intermediate-risk NMIBC. Post-operative continuous bladder irrigation (CBI) with saline (CSBI) or sterile water (CSWBI) is recommended as an alternative [3,6].

Despite its ability to prolong time to first recurrence and reduce the 5 yr recurrence rate from 59% to 45%, only one in six patients receives SI in the US [7,8,9,10]. In Sweden, only one in ten patients received SI during 2017–2023 [11]. Fear of side effects, cumbersome handling, and difficulty in identifying suitable patients have been presented as explanations for the underutilization of SI [12].

Twelve- to twenty-four-hour CBI with sterile water or saline matches a single Mitomycin C instillation in efficacy, incurs fewer adverse events, and costs less—yet it requires an overnight hospital stay [13,14]. A two-hour CBI might shorten hospitalization, but in the only trial to date it was inferior to mitomycin C, possibly because the study included CIS, recurrent NMIBC, and 50% high-risk tumours [15]. Recently, Onishi et al. showed in a randomized clinical trial that a three-hour CBI was non-inferior compared to overnight CBI [16].

Even though the EAU guidelines state that CBI is recommended as an alternative to SI mitomycin C, the optimal volume and duration remain uncertain, thus further research is needed. In 2018, Sundsvall Hospital’s urology department adopted a two-hour CSWBI protocol—based on those used at other Scandinavian centres—as a pragmatic postoperative treatment. Prior to 2018, no post-operative treatment was given.

The objective of this study was to compare two-hour CSWBI with no post-operative treatment, in a real-world setting, regarding safety and efficacy on the 12-month recurrence rate in primary NMIBC. By stratifying patients according to clinical risk factors, we aimed to assess efficacy across risk groups, thereby contributing to a risk-adapted pathway for personalized treatment.

## 2. Materials and Methods

### 2.1. Study Design and Setting

This retrospective before-and-after cohort study evaluated the impact of two-hour CSWBI on recurrence rates following TURBs of primary NMIBC in a real-world setting. The key exposure, CSWBI, was introduced in January 2018 as a new clinical routine for all TURB operations. Prior to 2018, TURB was performed without any peri- or post-operative treatment. Participants treated from 2014 to 2017 (no CSWBI) served as historical controls; those treated from the 2018 to 2021 received CSWBI. The study was conducted at the secondary-care urology department of Sundsvall Hospital, northern Sweden. All data were captured from electronic surgical and medical records retrospectively.

### 2.2. Participants

**Figure 1:**
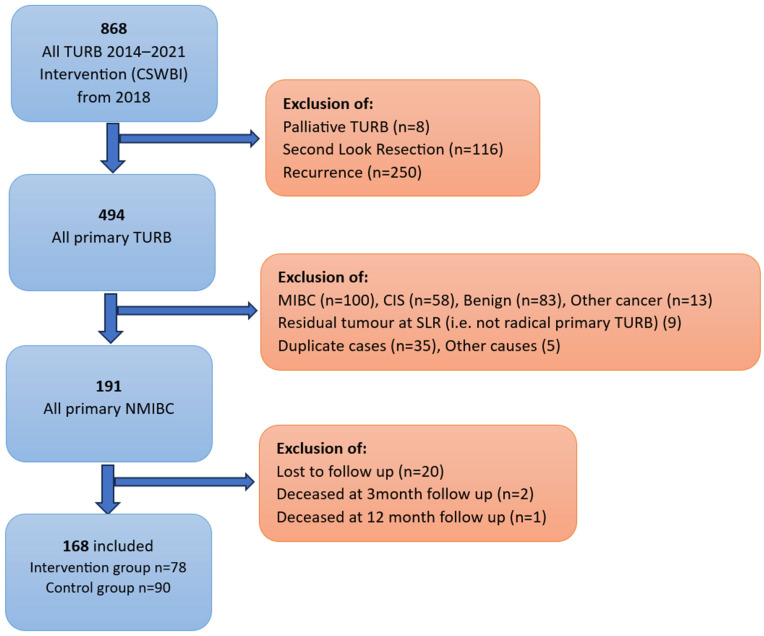
Inclusion-exclusion process. Medical records from all patients who underwent TURB between 2014 and 2021 were analyzed. All patients from 2018 onwards received post-operative CSWBI (intervention group). Prior to 2018 no post-operative treatment was given (control group). Inclusion or exclusion in the retrospective data analyses was based on indication for TURB and histopathology report. TURB = transurethral resection of the bladder. MIBC = muscle invasive bladder cancer. CIS = cancer in situ. SLR = second-look resection. NMIBC = non-muscle-invasive bladder cancer.

In the retrospective data extraction from clinical records all participants undergoing TURBs from 1 January 2014 to 31 December 2021 were eligible, except January 2018 when CSWBI was being implemented. We included all patients who underwent primary TURBs with a subsequent histological diagnosis of Ta or T1 NMIBC. Exclusions (shown in Figure 1) were made of non-primary TURBs, carcinoma in situ, muscle-invasive disease, benign disease, etc. Two participants in the CSWBI period who did not receive irrigation due to intraoperative perforation were reclassified into the control cohort.

### 2.3. Intervention and Variables

The key exposure in this study was the two-hour CSWBI, during which nine litres of sterile water were instilled via a three-way catheter, immediately following TURB. In most cases, the catheter was removed immediately after irrigation, except for a minority of participants who stayed overnight due to bleeding or other reasons. All TURBs were performed with a bipolar resectoscope.

Our primary outcome was tumour recurrence within twelve months, defined as either a visible lesion on follow-up cystoscopy confirmed by histopathology or the presence of malignant cells on bladder-wash cytology; cases in which a macroscopic recurrence was fulgurated without biopsy were also classified as recurrences.

Secondary outcomes included postoperative adverse events and hyponatremia. Adverse events were graded according to the Clavien–Dindo classification (CD) [17,18]. Minor perioperative perforations requiring no further intervention were classified as CD grade I. Hyponatremia was defined as serum sodium < 136 mmol/L, within one week of TURB, as recorded in the patient records but not assessed routinely in post-operative care.

In the planning stage we identified the following potential confounders: age, sex, smoking status, EAU risk group (accounting for tumour quantity, tumour size, tumour stage and grade), intravesical adjuvant (BCG or Mitomycin C), and surgeon experience. In Section 2.5 we specify which covariates were included in the final analysis and the rationale for not including all identified factors.

### 2.4. Data Sources and Measurement

Eligible participants were identified via the surgical database Orbit (Tieto Evry, Esbo, Finland). Using unique personal identification numbers, participants’ medical records were located, and data were extracted from them. The data extraction included baseline demographics, operative details, histopathology, adverse events, and recurrence data. Follow-up cystoscopy was planned according to guidelines [3,6], either quarterly or at 3 months and 12 months. In cases where the scheduled one-year cystoscopy was delayed, follow-up was accepted up to 14 months post-operatively to capture the intended one-year control. Data beyond this period were not retrieved as part of the predefined data collection for this cohort and were therefore not included in the analysis.

The timing of recurrence was defined as the time between primary TURB and recurrence-TURB or office fulguration.

### 2.5. Statistical Analysis

Statistical analysis was performed using RStudio (RStudio 2023.12.0+369 “Ocean Storm” Release) [19]. All variables were treated as categorical proportions except age and BMI which were treated as continuous variables. Patients were stratified according to EAU risk group classification 2021, defined elsewhere [20].

Primary and secondary outcomes were compared using Pearson’s Chi-squared tests for categorical variables and Wilcoxon rank-sum tests for continuous variables. To estimate the association between CSWBI and recurrence within twelve months, univariate and multivariable logistic regression analyses were performed, with results reported as odds ratios (ORs) with 95% confidence intervals (CIs) and *p*-values. Missing data was handled with complete-case analysis. The number of observations and events contributing to each model is reported.

The primary multivariable model was prespecified and adjusted for clinically relevant covariates, including EAU risk group, intravesical adjuvant therapy (BCG or Mitomycin C), sex, and age, in accordance with established recommendations regarding events per variable [21]. Adjustment for smoking status was planned but could not be performed due to a high proportion of missing data. To reduce overfitting and improve the events-per-variable ratio, surgeon experience was not included in the final multivariable model. No data-driven variable selection was performed.

Recurrence-free survival was illustrated using Kaplan–Meier curves, with time-to-event calculated from the date of TURBs. Recurrence, as defined above, was assigned to the date of surgical resection or office fulguration. Administrative censoring was applied at the time of the scheduled 12-month follow-up cystoscopy (allowing up to 15 months time-to-event in order to allow for time between follow up cystoscopy and TURB). Survival distributions between treatment groups were compared using the log-rank test.

We conducted a sensitivity analysis excluding early recurrences (within six months) to assess the robustness of the results under an alternative definition of recurrence, as these early recurrences may be influenced by incomplete initial resection or tumour characteristics associated with early recurrence.

Potential selection bias was mitigated by the study design with a temporal change in clinical routine for all patients going through TURBs. Confounding was mitigated by adjusting for known prognostic factors in multivariate models.

### 2.6. Ethics

This study was approved by the Swedish Ethical Review Authority (EPM 2022-06119-01, and EPM amendment 2023-08182).

## 3. Results

Baseline and clinical characteristics are shown in Table 1. A total of 168 participants were included in the final analysis: 78 in the intervention group and 90 in the control group (flow chart Figure 1). The median age was 73 years, 23% were female, 77% were male, and 74% were active or previous smokers.

There were a few differences between the groups regarding clinical characteristics. In the control group there was a higher proportion of tumours larger than 3 cm (control group n = 32 (40%) vs. intervention group n = 21 (28%)). In the intervention group there was a higher proportion of multifocal tumours (intervention group n = 34 (44%) vs. control group n = 26 (32%), and participants who received adjuvant Mitomycin C (n = 16 (21%) vs. n = 7 (8%)).

Risk group stratification according to the EAU 2021 model resulted in 19 low-risk patients (12%), 95 intermediate-risk patients (59%), 45 high-risk patients (28%), and three very-high-risk patients (2%). There were no statistically significant differences in risk group distribution between the treatment groups. Six participants could not be classified due to incomplete data regarding tumour size and focality; all belonged to the control group. Of these, four had T1G2–G3 tumours, two had TaG1–G2 tumours, and one experienced recurrence at the three-month cystoscopy (primary T1G2).

The use of adjuvant treatment differed between intermediate- and high-risk participants (with high- and very-high-risk grouped together). Among high-risk participants, 73% (n = 35) received BCG, whereas 34% (n = 32) of intermediate-risk patients received adjuvant treatment with either MMC or BCG.

### 3.1. Primary Outcome

**Figure 2:**
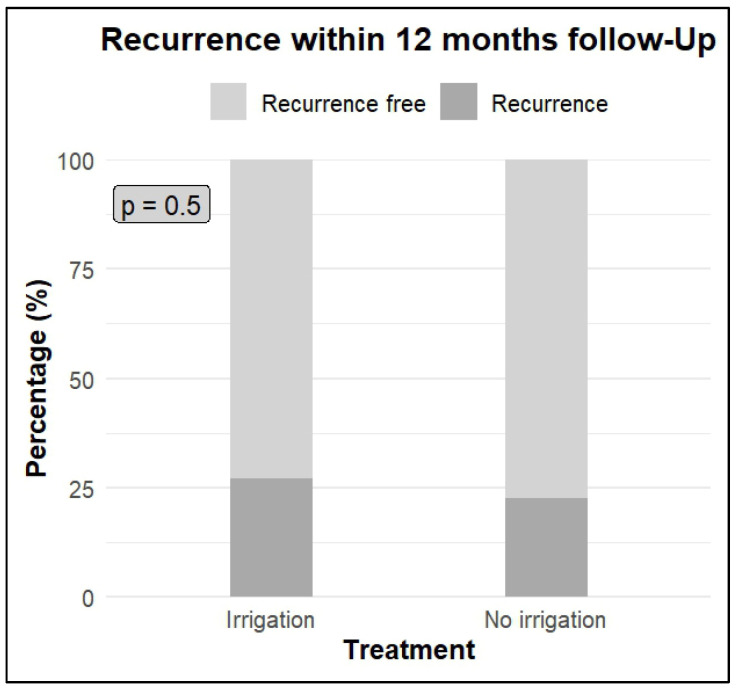
Primary endpoint—recurrence within 12 months follow up. There was no statistically significant difference in recurrence within twelve months between the groups (irrigation group 27% vs. 21% in the control group, *p*-value = 0.5).

The overall recurrence rate within twelve months was 24% (n = 40). There was no statistically significant difference between the treatment groups regarding recurrence within twelve months (intervention group 27%, n = 21, vs. control group 21%, n = 19, *p* = 0.5), as shown in Figure 2 and Table 2 along with cohort characteristics stratified by recurrence status within 12 months. One patient in the control group experienced progression from T1G3 (very-high-risk group) to muscle-invasive bladder cancer (MIBC) within three months and was therefore excluded from the twelve-month recurrence rate analysis.

**Figure 3:**
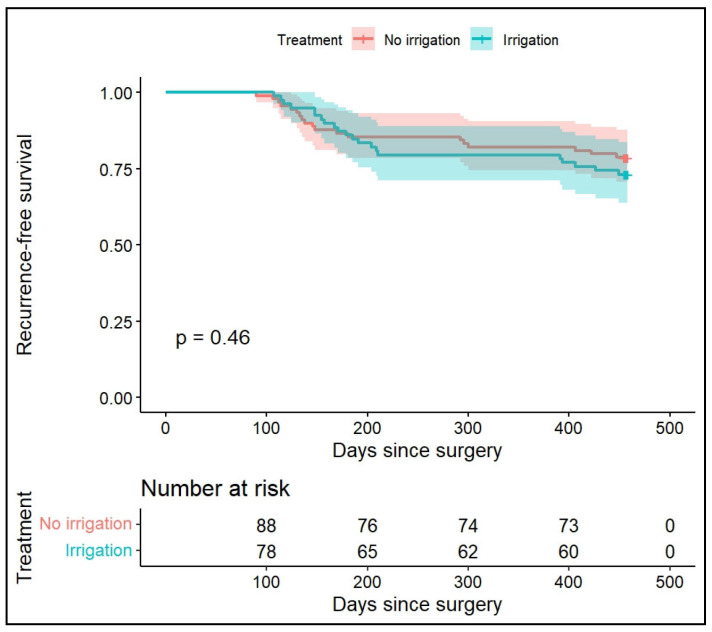
Kaplan–Meier curves illustrating recurrence-free survival within 12 months after TURB in patients receiving post-operative CSWBI or no irrigation. The curves show substantial overlap between groups (log-rank test, *p* = 0.46). Recurrences were detected at scheduled follow-up cystoscopies, and time-to-event was calculated from primary TURB to recurrence TURB or fulguration.

The Kaplan–Meier curves Figure 3 show substantial overlap between treatment groups, with no early separation and a non-significant log-rank test (*p* = 0.5).

Twelve-month recurrence outcomes stratified by EAU risk group and treatment are shown in Table 3 and are presented descriptively due to limited sample sizes within strata. Recurrence rates were similar among high-risk and intermediate-risk patients across treatment groups (recurrence rate 20–27%). A higher recurrence rate was observed among low-risk patients in the irrigation group compared with the control group (n = 2, 33% vs. n = 3, 23%); however, this finding should be interpreted with caution due to the small number of low-risk patients.

Logistic regression analysis for recurrence within twelve months showed no significant effect of irrigation when adjusted for EAU risk group, adjuvant treatment (BCG or MMC), sex, and age (OR 1.25, 95% CI 0.58–2.68, *p* = 0.6), as presented in Table 4.

Early recurrences (within first 6 months) were equal between groups and made up for approximately half of the recurrences within twelve months (intervention group 14%, n = 11, vs. control group 13%, n = 12, *p* > 0.9). Six out of these participants had recurrence again at the twelve-month follow-up, all of them were TaG1-G2 (intervention group n = 3, control group n = 3).

A sensitivity analysis was performed excluding participants with early recurrence (n = 23). Under this alternative definition, the 12-month recurrence rate was reduced by approximately half, while the relative distribution of recurrence between groups remained similar (intervention group 15%, n = 10, vs. control group 9%, n = 7, *p* = 0.3), see Table A1 in the Appendix A.

### 3.2. Secondary Outcome

**Figure 4:**
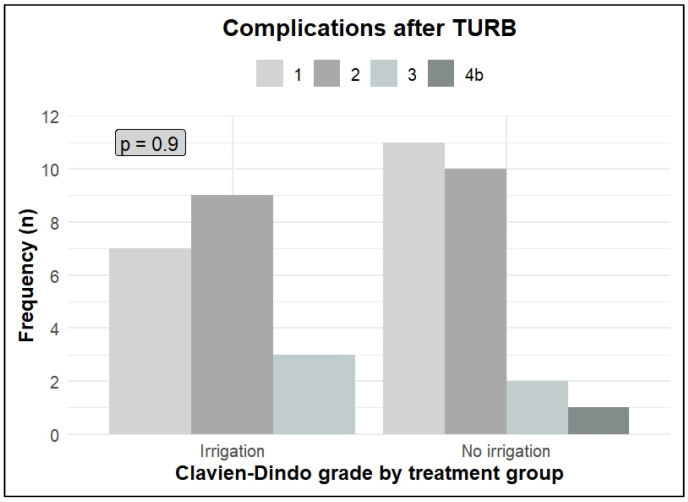
Complications after TURB +/− CSWBI. Post-operative complications were few, equally distributed in both treatment groups, and related to the TURBs. CSWBI = continuous sterile water bladder irrigation. TURB = transurethral resection of the bladder.

Adverse events (AEs) are presented in Figure 4. Post-operative adverse events according to Clavien–Dindo grades were evenly distributed between groups and were related to the TURBs. The most common AEs were haematuria, dysuria, and small bladder perforations requiring an indwelling catheter for a week. The grade 3 AEs (irrigation group, n = 3, vs. control group, n = 2) were severe haematuria requiring surgical intervention, and the grade 4 AE (n = 1) was a sepsis infection in the control group.

Hyponatremia (serum-sodium < 136 mmol/L) within a week after TURB was more common in the control group, although not statistically significant (19%, n = 10, vs. 9%, n = 5, *p* = 0.14, n = 106, n/a = 62). See Table 5.

## 4. Discussion

In this study, there was no statistically significant differences in recurrence rates within twelve months between post-operative two-hour CSWBI and no post-operative treatment after TURB for NMIBC. This finding remained unchanged after adjustment for relevant confounders, indicating no measurable benefit of short-duration CSWBI in this setting. These results are coherent with those from a recently published systemic review and meta-analysis [22].

Kaplan–Meier analysis demonstrated substantial overlap in recurrence-free survival between groups, with no early separation and a non-significant log-rank test. Interpretation of the curves is influenced by the predefined cystoscopic surveillance intervals used to detect recurrence, resulting in stepwise event patterns. In addition, time-to-event was calculated from the date of surgery, likely introducing a modest rightward shift, as recurrence was typically identified at cystoscopy prior to resection. As the primary estimand of interest was recurrence within 12 months, multivariable logistic regression was prespecified as the main inferential analysis.

Despite extensive research, recurrence in NMIBC remains a clinical challenge. The literature identifies five recurrence mechanisms: missed tumours during TURB, residual disease after TURBs, tumour re-implantation, drop metastasis from upper tract urothelial cancer, and the field change cancerization effect [23]. In preclinical mouse models re-implantation occurred within the first two hours after TURB [24], which explains why immediate post-operative treatment is usually more effective than delayed treatment [7].

Even though CSWBI was initiated immediately after TURB, there is a possibility that tumour re-implantation already had occurred, thus making tumour cells unavailable to the effect of CSWBI. It is also possible that two hours of irrigation is insufficient for hydrolysis to occur. Alternatively, continuous bladder irrigation might function primarily as mechanical flushing rather than inducing hydrolysis. In theory, manual bladder filling with sterile water could be a more effective approach, ensuring direct and prolonged exposure of tumour cells to sterile water.

Although somewhat counterintuitive, high-risk NMIBC showed better prognosis than intermediate-risk disease in this cohort. This may be explained by the fact that most high-risk patients received BCG, whereas only one third of intermediate-risk patients received adjuvant treatment. This pattern is consistent with larger datasets demonstrating superior recurrence-free survival among BCG-treated high-risk patients compared with intermediate-risk patients not receiving BCG [2]. The absence of effective immediate post-operative treatment, such as SI chemotherapy or overnight CBI, may also have contributed.

The one-year recurrence-free survival in the irrigation group was 73%, comparable to reports from studies of short-duration CBI (RFS 60–75%) [15,16,25], but lower than that reported for 12–24 h CBI (RFS 74–82%) and below predictions from EAU and EORTC risk models [2,13,14,20,26]. To our knowledge, Lenis et al. (2018) conducted the only other study evaluating two-hour CSBI, and our findings are consistent with theirs [15]. Although underpowered, both studies indicate that two-hour CSWBI does not confer a clinically meaningful reduction in recurrence rates, despite methodological improvements in the present study, including stricter exclusion criteria, standardized treatment, and a larger CSWBI cohort. 

Regarding safety, no statistically significant differences were observed between groups for adverse events (AEs) or hyponatremia, although conclusions regarding hyponatremia are limited by non-standardized testing and missing data, which may have introduced information bias. While serious adverse events were rare during the study period, two post-operative bladder ruptures occurred after study completion due to catheter misconnection. In light of these events and the absence of demonstrated benefit, CSWBI has been discontinued at our institution.

Our study had several strengths aimed at enhancing validity and minimizing bias. These included a quasi-experimental before–after design in which bladder irrigation was introduced from a specific date, allowing separation of cohorts over time and reducing selection bias through a uniform change in clinical practice. Follow-up was conducted according to guidelines, and loss to follow-up was limited to 10%.

At the same time, the retrospective single-centre design and modest sample size constitute important limitations. These factors resulted in some imbalance between groups and increased the risk of type II errors. Although such imbalances were addressed through multivariable regression adjustment for relevant prognostic factors, residual confounding cannot be entirely excluded. The before–after design is also potentially subject to temporal confounding; however, no relevant changes in clinical practice, surgical technique, patient selection, or follow-up routines occurred during the study period, apart from the introduction of CSWBI, and all patients were treated at the same urology clinic by the same group of surgeons.

Another limitation is the 12-month follow-up period. Although most NMIBC recurrences occur within the first year after TURBs [2,4], longer follow-up may capture additional late recurrences or progression events. While a sufficient number of recurrences had occurred by 12 months to allow meaningful comparison between groups, extended follow-up remains important for future prospective studies.

External validity is constrained by the single-centre setting; however, the clinic serves a population of approximately 240,000 inhabitants, comparable to other regional hospitals in Sweden and the Nordic countries. CSWBI was applied as routine clinical practice for four years, which we consider sufficient to assess its clinical performance. Our findings are consistent with the only other published study evaluating two-hour CBI and, despite limited power, collectively suggest that two-hour CSWBI does not confer a meaningful reduction in recurrence rates. The results are therefore likely applicable to similar Nordic centres considering short-term CSWBI as an alternative to SI intravesical chemotherapy for primary TURBs. Nevertheless, confirmation of these findings requires adequately powered prospective randomized controlled trials.

Future research should continue exploring the optimal duration of CBI, with the aim of identifying a short-term regimen that permits same-day surgery while overcoming barriers associated with both overnight CBI and IVC. Such an approach could enable more patients to benefit from perioperative adjuvant personalized treatment.

## 5. Conclusions

In this study post-operative two-hour CSWBI after TURB did not have a statistically significant effect on recurrence of NMIBC within twelve months, compared to no post-operative treatment.

## Figures and Tables

**Table 1:** Clinical characteristics of patients with primary NMIBC who underwent TURBs and received CSWBI (irrigation) or not.

Variable	N	Overall, n = 168	Irrigation, n = 78	No Irrigation, n = 90	*p*-Value ^1^
Sex, n (%)	168				0.2
Female		38 (23%)	14 (18%)	24 (27%)	
Male		130 (77%)	64 (82%)	66 (73%)	
Age (years), Median (IQR)	168	73 (68, 78)	74 (69, 78)	73 (67, 79)	>0.9
BMI, Median (IQR)	168	26.0 (24.3, 28.4)	26.1 (24.3, 29.3)	26.0 (24.2, 27.7)	0.2
Diabetes, n (%)	168	38 (23%)	18 (23%)	20 (22%)	>0.9
Prostate cancer, n (%)	128	27 (21%)	16 (25%)	11 (17%)	0.3
Missing data		40	14	26	
Smoking status, n (%)	112				0.4
Currently smoking		17 (15%)	9 (16%)	8 (15%)	
Non-smoker		29 (26%)	12 (21%)	17 (31%)	
Previous smoker		66 (59%)	37 (64%)	29 (54%)	
Missing data		56	20	36	
ASA-class, n (%)	166				0.7
1		22 (13%)	10 (13%)	12 (13%)	
2		83 (50%)	36 (47%)	47 (53%)	
3		60 (36%)	30 (39%)	30 (34%)	
4		1 (0.6%)	1 (1.3%)	0 (0%)	
Missing data		2	1	1	
Operation time, Median (IQR)	168	36 (24, 56)	40 (25, 57)	35 (21, 54)	0.4
Surgeon experience, n (%)	168				0.3
Consultant		103 (61%)	43 (55%)	60 (67%)	
Resident		29 (17%)	16 (21%)	13 (14%)	
Specialist		36 (21%)	19 (24%)	17 (19%)	
PDD (Hexvix), n (%)	168	55 (33%)	27 (35%)	28 (31%)	0.6
Tumour size, n (%)	155				0.12
<30 mm		102 (66%)	54 (72%)	48 (60%)	
≥30 mm		53 (34%)	21 (28%)	32 (40%)	
Missing data		13	3	10	
Tumour quantity, n (%)	158				0.2
Multifocal		60 (38%)	34 (44%)	26 (32%)	
Single		98 (62%)	44 (56%)	54 (68%)	
Missing data		10	0	10	
Stage/Grade, n (%)	168				0.2
T1G2		8 (4.8%)	2 (2.6%)	6 (6.7%)	
T1G3		32 (19%)	11 (14%)	21 (23%)	
TaG1		67 (40%)	32 (41%)	35 (39%)	
TaG2		47 (28%)	24 (31%)	23 (26%)	
TaG3		14 (8.3%)	9 (12%)	5 (5.6%)	
EAU Risk Group, n (%)	162				0.08
Very high		3 (1.9%)	0 (0%)	3 (3.6%)	
High		45 (28%)	20 (25%)	25 (30%)	
Intermediate		95 (59%)	52 (67%)	43 (51%)	
Low		19 (12%)	6 (7.7%)	13 (15%)	
Missing data		6	0	6	
BCG, n (%)	168	52 (31%)	23 (29%)	29 (32%)	0.7
Mitomycin C, n (%)	168	23 (14%)	16 (21%)	7 (8%)	0.017
MitomycinC + BCG, n (%)	168	3 (1.8%)	0 (0%)	3 (3.3%)	0.2

Patients treated at the urology department of Sundsvall Hospital 2014–2021. NMIBC = non-muscle- invasive bladder cancer. CSWBI = continuous sterile water bladder irrigation. IQR = interquartile range. BMI = body mass index. ASA = American Society of Anesthesiologists physical status classification. PDD = photo dynamic diagnostics. Hexvix = Hexaminolevulinate. EAU Risk Group 2021 classification. BCG = Bacillus Calmette Guerin. ^1^ Pearson’s Chi-squared test; Wilcoxon rank-sum test; Fisher’s exact test.

**Table 2:** Cohort characteristics stratified by recurrence status within 12 months.

Variable	N	Overall, N = 167 ^2^	Recurrence, N = 40	Recurrence Free, N = 127	*p*-Value ^1^
Treatment, n (%)	167				0.5
Irrigation		78 (100%)	21 (27%)	57 (73%)	
No irrigation		89 (100%)	19 (21%)	70 (79%)	
Sex, n (%)	167				0.2
Female		37 (100%)	12 (32%)	25 (68%)	
Male		130 (100%)	29 (22%)	101 (78%)	
Age (years), Median (IQR)	167	73 (68, 79)	71 (66, 78)	74 (68, 79)	0.4
Smoking status, n (%)	111				0.4
Currently smoking		17 (100%)	3 (18%)	14 (82%)	
Non-smoker		29 (100%)	10 (34%)	19 (66%)	
Previous smoker		65 (100%)	16 (25%)	49 (75%)	
Missing data		56	11	45	
BMI, Median (IQR)	167	26.0 (24.3, 28.4)	26.0 (24.5, 28.4)	26.0 (24.1, 28.6)	0.7
Surgeon, n (%)	167				>0.9
Consultant		102 (100%)	23 (23%)	79 (77%)	
Resident		29 (100%)	8 (28%)	21 (72%)	
Specialist		36 (100%)	9 (25%)	27 (75%)	
PDD (Hexvix), n (%)	167	54 (100%)	14 (26%)	40 (74%)	0.7
Tumour size, n (%)	154				0.3
<30 mm		102 (100%)	23 (23%)	79 (77%)	
≥30 mm		52 (100%)	16 (31%)	36 (69%)	
Missing data		13	1	12	
Tumour quantity, n (%)	157				0.1
Multifocal		59 (100%)	19 (32%)	40 (68%)	
Single		98 (100%)	20 (20%)	78 (80%)	
Missing data		10	1	9	
PAD, n (%)	167				0.3
T1G2		8 (100%)	2 (25%)	6 (75%)	
T1G3		31 (100%)	3 (10%)	28 (90%)	
TaG1		67 (100%)	19 (28%)	48 (72%)	
TaG2		47 (100%)	12 (26%)	35 (74%)	
TaG3		14 (100%)	4 (29%)	10 (71%)	
EAU risk group, n (%)	161				>0.9
Very high		2 (100%)	0 (0%)	2 (100%)	
High		45 (100%)	10 (22%)	35 (78%)	
Intermediate		95 (100%)	24 (25%)	71 (75%)	
Low		19 (100%)	5 (26%)	14 (74%)	
Missing data		6	1	5	
BCG, n (%)	167	52 (100%)	12 (23%)	40 (77%)	0.9
MitomycinC, n (%)	167	23 (100%)	9 (39%)	14 (61%)	0.066

^1^ Pearson’s Chi-squared test; Wilcoxon rank-sum test; Fisher’s exact test. ^2^ n = 167 because of 1 patient in the control group underwent cystectomy due to progression of disease at 3 months follow-up. IQR = interquartile range. BMI = body mass index. PDD = photo dynamic diagnostics. BCG = Bacillus Calmette Guerin.

**Table 3:** EAU risk group distribution and 12-month outcome stratified by treatment.

	Irrigation			No Irrigation		
Characteristic	N	Recurrence ^1^	Recurrence Free n (%)	N	Recurrence ^1^	Recurrence Free n (%)
EAU risk group	78			83		
Very high		0 (-)	0 (-)		0 (0%)	2 (100%)
High		5 (25%)	15 (75%)		5 (20%)	20 (80%)
Intermediate		14 (27%)	38 (73%)		10 (23%)	33 (77%)
Low		2 (33%)	4 (67%)		3 (23%)	10 (77%)

^1^ Total n = 161. Excluded from analysis; six patients were not classifiable according to EAU risk groups; one very high-risk patient in the no-irrigation group progressed at three months and underwent cystectomy. Descriptive statistics only.

**Table 4:** Logistic regression for recurrence within 12 months follow-up.

Characteristic	N ^1^	Crude OR ^1^	95% CI ^1^	*p*-Value	N	Adjusted ^2^ OR	95% CI ^1^	*p*-Value
Treatment	167				161			
No irrigation	19	(Ref.)	-		18	(Ref.)	-	
Irrigation	21	1.36	0.67–2.78	0.4	21	1.25	0.58–2.68	0.6

^1^ N = number of observations used in the regression model, OR = odds ratio, CI = confidence interval. ^2^ Adjusted for EAU risk group, adjuvant treatment (BCG or MMC), sex, and age. Complete-case analysis in the adjusted model rendered n = 161 due to six participants not EAU risk classifiable, out of which one was recurrence in the control group.

**Table 5:** Hyponatremia within one week after treatment.

Variable	N	Overall, N = 168 ^1^	Irrigation, N = 78 ^1^	No Irrigation, N = 90 ^1^	*p*-Value ^2^
Hyponatremia	106				0.2
Eunatremia *		91 (86%)	48 (91%)	43 (81%)	
Na < 136 mmol/L		15 (14%)	5 (9.3%)	10 (19%)	
Missing data		62	25	37	

^1^ n (%). ^2^ Pearson’s Chi-squared test. * Eunatremia was defined as serum-sodium 136–144 mmol/L. No statistically significant difference was observed between groups. Sodium levels were not measured routinely.

## Data Availability

The raw data supporting the conclusions of this article will be made available by the authors on request.

## Abbreviations

The following abbreviations are used in this manuscript:

NMIBC	Non-Muscle-Invasive Bladder Cancer
SI	Single Immediate post-operative intravesical instillation of chemotherapy
CSWBI	Continuous Sterile Water Bladder Irrigation
CSBI	Continuous Saline Bladder Irrigation
CBI	Continuous Bladder Irrigation
TURB	TransUrethral Resection of the Bladder
EAU	European Association of Urology
CD	Clavien–Dindo classification of adverse events
EORTC	European Organisation for Research and Treatment of Cancer
BMI	Body Mass Index
OR	Odds Ratio
CI	Confidence Interval
IQR	InterQuartile Range
MIBC	Muscle Invasive Bladder Cancer
CIS	Cancer In Situ
SLR	Second Look Resection
BCG	Bacillus Calmette-Guérin
MMC	Mitomycin C
RFS	Recurrence Free Survival

## Appendix A

**Table A1:** Sensitivity analysis. Recurrence within 12 months after excluding early recurrences.

		Overall N = 144	Irrigation N = 67	No Irrigation N = 77	*p*-Value ^1^
Follow up 12 months, n (%)					0.3
Recurrence		17 (12%)	10 (15%)	7 (9%)	
Recurrence free		127 (88%)	57 (85%)	70 (91%)	

^1^ Pearson’s Chi-squared test. Sensitivity analysis of primary endpoint, excluding participants with recurrence within six months (n = 23). One participant suffered progression at 3 months and was not included in the recurrence analysis.
